# Prevalence of Human Papillomavirus (HPV) and HPV Vaccination Completion Rates Among Gay and Bisexual Men in Dar es Salaam, Tanzania

**DOI:** 10.3390/venereology4010001

**Published:** 2024-12-25

**Authors:** Lucy R. Mgopa, Ever Mkonyi, Mabula Kasubi, Alan G. Nyitray, Michael W. Ross

**Affiliations:** 1 Department of Psychiatry and Mental Health, School of Clinical Medicine, Muhimbili University of Health and Allied Sciences, Upanga Campus, Dar es Salaam P.O Box 65001, Tanzania;; 2 Medical Education Research and Evaluation, Case Western Reserve University, Cleveland, OH 44106, USA;; 3 Muhimbili National Hospital, Dar es Salaam P.O. Box 65000, Tanzania;; 4 Center for AIDS Intervention Research (CAIR), Department of Psychiatry and Behavioral Medicine, Medical College of Wisconsin, Milwaukee, WI 53202, USA;; 5 Institute of Sexual and Gender Health, Department of Family Medicine, University of Minnesota, Minneapolis, MN 55454, USA

**Keywords:** HPV, gay and bisexual men, anal, vaccination, Africa, Tanzania, return rate

## Abstract

High-risk Human Papillomavirus (HPV) is a sexually transmissible virus that causes cancer. Vaccination against HPV is available up to age 45. Gay and bisexual men (GBM) are at high risk for oropharyngeal and anal cancers caused by HPV. As part of a larger study of HPV prevention in GBM, we obtained anal swabs for HPV and offered HPV vaccination to GBM in Dar es Salaam, Tanzania. Participants were recruited by an outreach worker experienced with the GBM subculture using Respondent-Driven Sampling (RDS) from seeds. Eighty-three of a possible one hundred participants (mean age 26) were enrolled, tested, and given a first vaccination dose. Anal swabs were tested for twenty-eight HPV genotypes, both high and low risk for carcinogenicity, and a median of seven different HPV genotypes was found in participants. A total of 87% of participants carried at least one HPV genotype, and 76% carried at least one high-risk genotype. As a result of harassment and unanticipated risk to participants and staff at the unmarked community-based site, this study was terminated before the sample size was reached. Since a full course of vaccine was the standard of care, participants were contacted using the contact method they had provided to arrange follow-up vaccination doses at an alternative clinical site. Twenty-nine percent received the additional vaccination. A further 6% made appointments but did not attend, and another 10% would have received the second vaccination if they were incentivized to attend. The rate of HPV in anal samples was significantly higher than in our study using the same recruitment method in the same city in 2011–2012. The HIV rate was 45%. Anal HPV rates, including high-risk HPV rates, were significantly higher than in a similar population of gay and bisexual men 12 years ago. It is possible to provide HPV vaccination to gay and bisexual men in Tanzania and have approximately 45% potentially re-attend for a second dose if they are incentivized. Great care must be used to situate vaccination to avoid stigma leading to harassment, especially where homosexuality is criminalized. We infer that the high-risk harassment faced by participants and lack of incentive for the second visit depressed the return rate for revaccination.

## 1. Background

Anal Human Papillomavirus (HPV) infection, often in conjunction with HIV infection, has been reported at high rates in gay and bisexual men (GBM) in several Sub-Saharan African countries, including our previous studies in Tanzania [1,2].

Recently, Lorway et al. [3] called for the urgent provision of HPV vaccines to men who have sex with men (MSM). This followed their experience in Kenya where the HPV vaccine is licensed for use among males and females, but health officials have indicated that such vaccines are earmarked only for females. In addition to noting the data on extensive HPV infection in MSM and their high risk of anal cancer in Sub-Saharan Africa (SSA), Lorway et al. [3] urge the expansion of vaccine access to this vulnerable and stigmatized group.

There is extensive evidence of high rates of anal HPV infection in MSM in SSA. Our 2011–2012 study of Tanzanian MSM in Dar es Salaam [1,2] found that anal hr-HPV infection was 54.3%, and HPV-16 was 17.2%. Mbouma Bourassa et al. [4] reported on 42 MSM in Bangui, Central African Republic, in 2019, where the prevalence of anal HPV was 69.1%, and hr-HPV was 57.1%. Data obtained from 350 Rwandan MSM [5] indicated 20.1% had anal hr-HPV infection, and penile hr-HPV prevalence was 35.0%. The prevalence of HPV-16 and HPV-18 was not significantly different for anal and penile sites. West African data are similar, with Koyalta et al. [6] finding 70% with any HPV and 56% with hr-HPV in 2019 in Bamako, Mali (of which HPV-16 was 24%). In Togo, 207 MSM in four cities [7] had an HPV anal infection rate of 52.7% and hr-HPV of 44.9%. Studying MSM in four West African countries in 2021, Yaya et al. [8] reported that HIV infection was significantly positively associated with anal HPV infection (OR = 3.61, 95% CI 2.48–5.27), a finding first reported by Nowak et al. in Nigeria in 2016 and subsequently replicated [9,10]. In the West African data, Yaya et al. [8] calculated that with 9-valent HPV vaccination, the prevalence of HPV infection would be reduced by 34.6% in HIV-negative men and by 54.9% in HIV-positive men. To the best of our knowledge, HPV vaccination of MSM in SSA has not, however, been successfully attempted.

HPV types 16 and 18 are associated with 85% of HPV-related head and neck cancers and 87% of all anal cancers, so vaccination of males is as important as in females, as is vaccination of people who are HIV infected [10].

**Figure 1.**
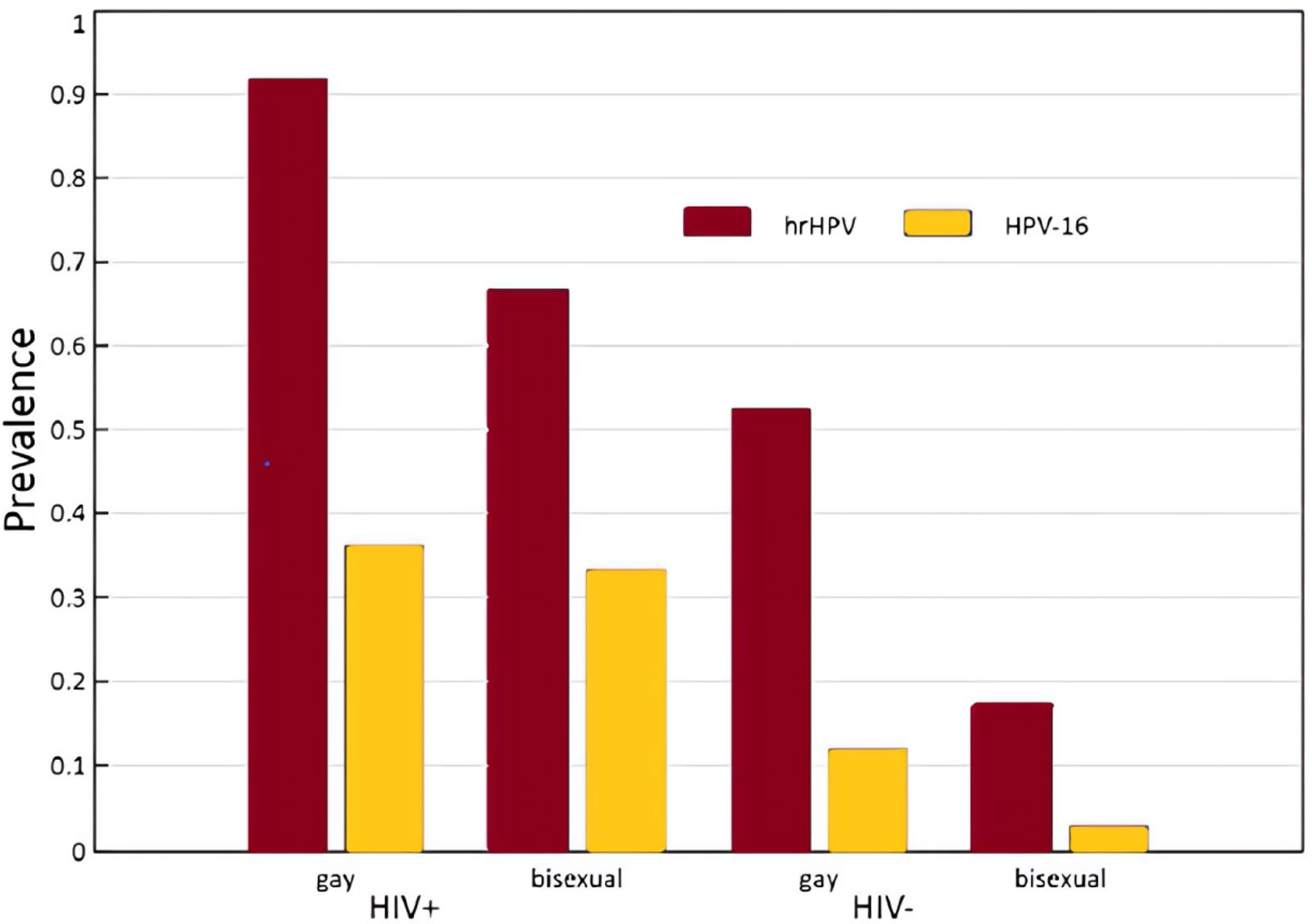
HPV-16 and high-risk HPV prevalence by sexual identity and HIV in gay and bisexual men in Dar es Salaam, 2011–2012.

Twelve years after our study of MSM in Tanzania [1,2] (Figure 1 illustrates those HPV data [2]), we again recruited MSM in the same city and used the same methods to ascertain the rate of anal HPV infection, provide education about HPV-associated cancers in MSM, and determine response (and return for a second vaccine dose) to offering HPV vaccination to these men. Return for additional doses is important to assess the proportion completing multiple-dose HPV vaccine regimens as a vaccine completion measure.

## 2. Methods

Respondent-Driven Sampling recruitment for our previous anal HPV study in 2011–2012 in Dar es Salaam, Tanzania [2], was replicated, and in addition, we offered free and incentivized HPV vaccination to participants.

Recruitment was coordinated with a community outreach worker. The approved sample size was 100 based on our previous study of anal HPV in 116 GBM in Dar es Salaam in 2011–2012 [2]. Sample size calculation was based on being able to replicate the findings from the first study [1] with a similar population size and proportion at *p* < 0.01. Given the stigmatized and criminalized nature of homosexuality in Tanzania, MSM were recruited using RDS, which provided access to MSM utilizing established networks of friends and acquaintances. Three initial acquaintances, covering a range of ages and education levels, were contacted by a community outreach worker in Dar es Salaam, and these persons referred up to 3 persons each using study coupons. These men in turn recruited additional MSM in a similar iterative fashion with a target of 100 enrollments. The study site was a house that was being used as a rented office in a central commercial suburb, where the premises were secured with a solid high fence and gate, ensuring private entry and exit. This arrangement was successful in the previous study [1,2] in a different area of the same suburb.

The survey for data collection was uploaded online, and eligible participants consented and filled out the baseline survey at the study office using a laptop or tablet. All data were directly recorded on a firewalled and password-protected US (University of Minnesota) cloud site. Participants received in-person training to digitally detect anal potentially cancerous lesions using the clinical trial training described by Nyitray et al. [11] and using an identical pelvic manikin training model. A dry Dacron swab was used to self-collect exfoliated cells from the anal canal by participants after demonstration and collection by the clinicians using the training manikin. This swab was immersed in a specimen transport medium (Hologic, San Diego, CA, USA) and placed into −20 °C storage at the Central Pathology laboratory at the Muhimbili National Hospital until testing. Anal swabs obtained were tested for HPV genotypes using Cobas^®^ 4800 (Roche Molecular Systems, Inc., Branchburg, NJ, USA).

All participants chose to receive the vaccinations, which were Cervarix^®^ (bivalent HPV-16, 18) vaccines. Contact details for the second vaccine dose were provided by participants. The Tanzanian National Immunization Program recommended [12] a two-dose vaccination 6 months apart. However, the National Program only considers girls aged between 9 and 14 as vaccine targets. The incentive to participate in this study visit was TZS 50,000 (~USD 20.00). Following unexpected pursuit at the site during data collection, this study was stopped after one month, at the Tanzanian and US PIs’ request, due to this unanticipated risk to participants and staff. As the risk level remained high, this study was subsequently formally closed. All IRB approvals included the provision of subsequent vaccine doses if this study terminated or participants withdrew as the standard of clinical care. Contact was made, or attempted, with all participants for a free second vaccine dose in a place of their choice. This was performed to complete the appropriate medical standard of care. However, no research incentive was provided, as this study was closed at the time of the second vaccination.

### 2.1 Vaccine

Cervarix^®^ (bivalent HPV-16,18) is licensed by the manufacturer (GlaxoSmithKline, Wavre, Belgium) for girls and boys aged 9–14 years as a 2-dose schedule (5–13 months apart). If the recipient’s age is ≥15 years at the time of the first dose, the manufacturer recommends that three doses should be given (at 0, 1–2.5 months, and 5–12 months) [10].

### 2.2 Sample Collection and Storage

This pilot study analyzed a final sample (*n* = 83) of provider-collected anal swabs to determine the prevalence of high-risk oncogenic HPV genotypes among men in Tanzania. The collected anal swabs were immersed in 200 μL of ATL lysis buffer from the QIAamp DNA Mini kit (Qiagen, Hilden, Germany; cat. 51304) and stored at 4–8 °C before DNA extraction.

### 2.3 Nucleic Acid Extraction

Anal swabs in ATL buffer were vortexed at full speed, and a 200 μL of fecal mix was aliquoted for DNA extraction using the QIAamp DNA Mini kit (Qiagen, Hilden, Germany; cat 51304) following the manufacturer’s protocol for isolation of DNA. Extracted DNA samples were eluted in 50 μL of elution buffer and stored at −20 °C until PCR analysis. Extra care was taken during the extraction of these samples to avoid cross-contamination by simultaneously working on a few samples at a time and continuously changing working gloves. HPV detection was performed with Anyplex28. In two separate wells, Anyplex28 detects 28 HPV types into two groups, namely, Panel A (16, 18, 31, 33, 35, 39, 45, 51, 52, 56, 58, 59, 66, and 68) and Panel B (6, 11, 26, 40, 42, 43, 44, 53, 54, 61, 69, 70, 73, and 82), as well as an internal control (IC) for each panel. As a semi-quantitative assay, Anyplex28 uses dual priming oligonucleotide (DPO) and tagging oligonucleotide cleavage and extension (TOCE) technologies. This kit reports the typing results with a crossing point (Cq) range of ≤31 cycles (+++), 31–39 cycles (++), and >40 cycles (+) (hereafter, signal strength or Seegene score). A sample was deemed “invalid” if it had a negative IC or an IC with a Cq > 40. To assess the samples for the presence of HPV, 5 μL of extracts was added to 15 μL of PCR master mix, and this was tested on Anyplex28 according to the manufacturer’s instructions using a CFX96 Real-time PCR detection system (Bio-Rad, Hercules, CA, USA).

### 2.4 Statistics

This was a descriptive analysis. HPV prevalence and participant baseline characteristics were reported as %, means, standard deviations, medians, and interquartile ranges. We initially intended to carry out similar comparisons to the 2011–2012 Dar es Salaam study and compare those participants with vs. without oncogenic HPV genotypes. However, the data from the present study showed only 11 participants without oncogenic HPV infection, a number which has insufficient power to carry out quantitative testing between infected and uninfected groups. Comparisons between 2011–2012 and the present (2023) samples were carried out using N-1 χ^2^ tests (which are preferable with smaller sample sizes [13]) in SocialScienceStatistics 2024. Significance levels were set at *p* < 0.01.

## 3. Results

**Figure 2.**
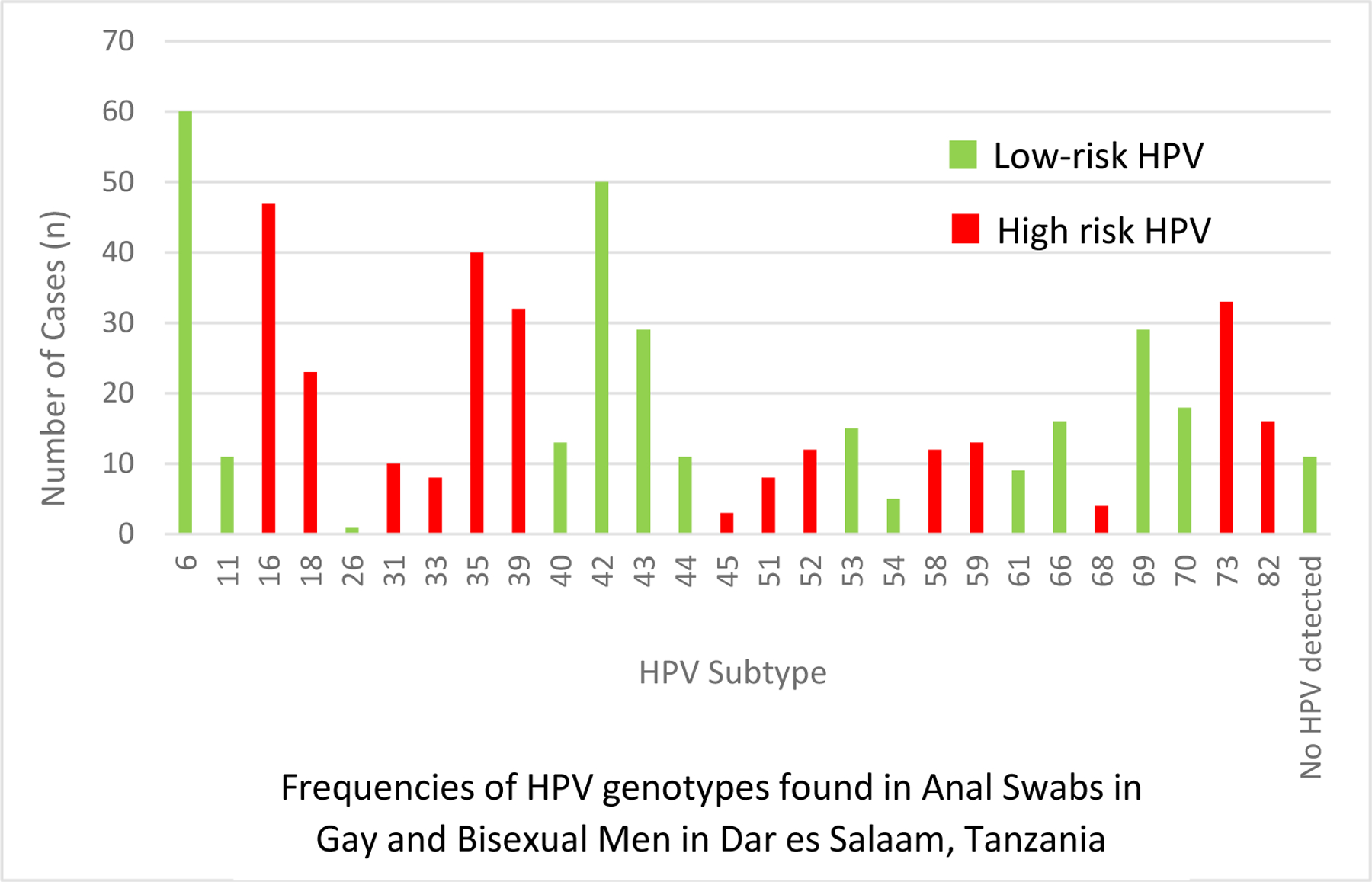
Frequencies of HPV genotypes tested.

This study, at the point it was halted, included 83 participants who were assigned male at birth. Of these, sixty-six identified as gay men, fourteen as bisexual, and three as transgender women. Data are presented in Figures 1 and 2 and Tables 1 and 2. The majority of participants were either dating but not living with a partner (40%) or single (35%), followed by those dating and living with their partner (18%). A smaller group (7%) included participants who were separated, married to a woman, living with a woman, or widowed. Five participants did not respond to the relationship status question.

In terms of annual take-home income, 31% of participants earned less than TZS 50,000 (approximately USD 20), 7% earned between 50,001 and 150,000, 10% earned between 151,000 and 300,000, 4% earned between 300,001 and 450,000, and 33% earned more than 450,001. Additionally, 16% of participants chose not to disclose their income.

When asked about their number of lifetime sexual partners, the distribution was as follows: twenty-eight participants reported having 1–10 partners, twelve had 11–20 partners, twenty-one had 21–100 partners, nine had 101–1000 partners, and six reported having more than 1000 partners. We collapsed the reported responses for >10,000 partners because of small cell sizes and because at this point these two categories were presumed to be a participant estimate for a very large number of sexual numbers rather than an exact count.

Regarding education level, most participants had a middle school education (60%), followed by those with less than a high school education (17%). Additionally, 7% had a high school education, and 12% had either some college education or a Bachelor’s degree. The education level of this sample was considerably higher than in the general male population in Tanzania. High-risk HPV was detected in 75.9% of participants in the present study, including HPV-16 in 47 and HPV-18 in 23 (Figure 2). HPV of either high or low risk was detected in 72 (86.8%) of the anal swabs. The most common hr-HPV genotypes were 16 (56%), 32, 39, 73, and 37. Nearly half (44.5%) of participants reported they were HIV positive.

To enable comparison with our 2011–2012 GBM sample in Dar es Salaam (Figure 1), we also analyzed the 2023 data using only 14-genotype hr-HPV markers, as used in the 2011–2012 data analysis. We found the same 75.9% infected in the 2023 data when only using the 14-genotype analysis used in 2011–2012. This finding of 75.9% infected in 2023 compares with 54.3% in the 2011–2012 sample [1].

The median number of different genotypes detected in 2023 was seven (interquartile range = 4, range 0–12). Only eleven (13.3%) had no evidence of HPV infection (high or low risk), and only four (4.8%) had a single genotype detected. In three of the four, this was HPV-16.

The difference between the proportion of participants with hr-HPV infection between the 2011–12 sample and the present 2023 sample using an N-1 χ^2^ test for differences between proportions was a difference of 21.6% (95% CI 8.13–33.65%), χ^2^ = 9.67, df = 1, and *p* = 0.002.

There was no significant difference in HIV proportions between the two samples using the N-1 χ^2^ test for difference between proportions (difference 10.9%, 95% CI −2.71–24.23%, χ^2^ = 2.42, df = 1, *p* = 0.12).

All participants consented to the first dose. No participants reported adverse biological events following vaccination. After this study was halted, vaccination for the second dose was offered to all participants for free as per the IRB protocol; 48.3% were uncontactable via the contact method specified by them (a mobile phone number or email or contact through the community outreach worker) or did not return contact requests. At least two attempts were made to contact all participants. The 51.3% who were contactable included twenty-four (28.9%) who received the second vaccine dose in a clinic administered by the clinician; five made appointments but did not attend, three had died (one as result of an accident, two of AIDS-related illness), and one had moved to a neighboring country. One (transgender, assigned male at birth) participant had visibly feminized as a result of hormone treatment and was afraid to go out in public, and eight required an incentive from TZS 30,000 to 50,000 to attend.

## 4. Discussion

There was a high rate (>75%) of hr-HPV in gay and bisexual men recruited in Dar es Salaam, Tanzania (Figure 2). This significantly exceeds by 26% the rate found using an identical RDS sampling method in the same city and the same suburbs in the city in 2011–2012. These data suggest that anal HPV infection rates have risen and remain extremely high in this population, although we cannot make a direct comparison between these relatively small samples, and the results are suggestive only. We are not aware of previous studies in SSA where anal HPV rates in MSM have been compared over time. As in 2011–2012, these HPV rates are associated with a high level of anal HIV infection. This is unsurprising given that risk behaviors for both include receptive anal sex.

The hr-HPV detected are consistent with penile hr-HPV genotypes detected in penile swabs in Tanzania by Olesen in 2013 [14] (types 16, 18, 35), although they did not ask about sexual behavior to distinguish MSM in that study. They are also consistent with genotypes reported by Seyoum in a meta-analysis in East Africa (16, 18, 39) [15], Mayaud [16] in HPV-positive cervical specimens in women in Northwest Tanzania (genotypes 16, 18), and Dartell in 2012 in cervical specimens in over 3000 Tanzanian women [17]. Given the rates of sex work in MSM in East Africa, whose clients are often non-homosexually identified bisexual men (studies in Mombasa, 200 miles north of Dar es Salaam, report that at least 739 (95% CI 690–798) men were selling sex to other men in that city) [18], we would expect Dar es Salaam, at nearly five times the size of Mombasa, to be similar) [19]. From a public health perspective, MSM populations in Tanzania may also be bridging to both male and female populations. Our 2011–2012 data indicated that a majority of MSM sampled using RDS in Dar es Salaam who participated had experience with sex work [19].

In Tanzania, male–male sex is criminalized, with penalties ranging from 5 years imprisonment to life. In this context, studying and providing health services to MSM meets some challenges when it becomes publicly known. Such challenges and risks to the safety of both participants and staff led to stopping the research study in consultation with our IRB when only 83% of the approved sample number had been enrolled. However, as per our IRB approvals, we provided the subsequent dose of HPV vaccination free as the clinical standard of care. This unexpected termination of this study due to unforeseen risks provided a serendipitous opportunity to observe clinical data on return for the best standard of care vaccinations, which were free but where no incentive could be provided given the closure of this study.

The 29% return rate for the second vaccine dose is difficult to compare with the return rates for HPV vaccination in girls in East Africa. Wigle et al. [20] note that when returning for a second vaccination for girls at a vaccinable age (9–14), parental response is one of the most important factors, along with the fact that girls are accessed and enrolled in school and vaccinated while at school [12,21]. Neither of these factors applies to our MSM sample.

However, if we add the requirement of an incentive to our return rate, nearly 40% of our sample could be vaccinated, with the second dose provided if there was an incentive. We believe that the experience of hostility and resulting fear in our sample depressed returning for a second dose, although we cannot quantify that. The re-contact details that needed to be provided may also have concerned participants, although recording all data on the password-protected and firewalled US university website rather than a local one was used as a protective mechanism.

However, given the fact that homosexuality and same-sex sexual behavior are criminalized in Tanzania, with substantial prison time penalties, it is clear that healthcare provision towards this key population remains a problem. Despite having Tanzanian IRB and government support for research work involving HIV and other STIs among the most vulnerable populations, like GBM, harassment and potentially worse are probable, as our experience demonstrated. On one occasion, a few days before the harassment commenced, some study participants gathered on the street outside the study site and engaged in loud and flamboyant behavior, which led to complaints to the study staff from several business owners in the street.

Data protection was built into the study design. Medical and study records may be subpoenaed in a criminal matter, considering that homosexual behavior is a criminal offense in Tanzania. Our study had an NIH Certificate of Confidentiality, which applies only to the US and has no necessary impact in other jurisdictions. This was a factor in our decision to collect identifiable data directly to a firewalled and password-protected US website. We conclude, based on our experience, that despite existing and continuous efforts towards reducing STI infections by the Tanzanian Government through the Ministry of Health, legal disparities existing towards issues related to homosexuality/same-sex attraction are a major barrier to HPV vaccination in sexual minority populations.

This study adds empirical experience to Lorway et al.’s [3] concerns in an adjacent East African country. We recruited more than 80% of the MSM sample in under 3 weeks; all consented to receive the HPV vaccine doses, suggesting that this population was willing to engage in HPV vaccination studies.

While Kreimer et al. [22] reported that 10 years after HPV vaccination in women single-dose vaccine efficacy against HPV16 or 18 infections remained high and HPV16 or 18 antibodies remained stable, they suggest that a single dose of a bivalent HPV vaccine may induce sufficient durable protection and that additional doses may be unnecessary. However, we planned multiple doses in this study since it was the clinical standard of care for people aged ≥ 15 years in Tanzania; at the time, this study was approved by both IRBs, and participants consented to multiple doses. Subsequently, in 2024, the WHO and its partners, including the Tanzanian Ministry of Health, announced a single dose as the standard of care for HPV vaccination for girls aged 9–14 [23].

Given our experiences, it may be appropriate to consider whether single HPV vaccine doses may also be suitable for adults, including MSM, in SSA. On the other hand, the high co-prevalence of HIV and HPV in GBM samples in this region may raise the importance of providing an additional dose to potentially immunosuppressed individuals. An additional issue is that HPV vaccination will not be effective for genotypes that have already infected the individual, and in this sample, with a mean age of 26, the median number of HPV genotypes that participants have already been infected with was seven, with over 75% being high-risk genotypes. These data suggest that if HPV vaccination of gay and bisexual men in SSA is undertaken, it should be performed at the earliest possible time before or shortly after sexual debut. Given these data, a high-valent vaccine would be most appropriate, particularly if an individual is not infected with the vaccine genotypes. However, the time and cost of determining what genotypes the participant is infected with would be difficult to justify, especially given return rates for follow-up. Given that up to 90% of anal and oropharyngeal carcinomas are caused by types 16 and 18, a high valence vaccination may not be cost effective.

### Limitations

There are several limitations. First, while we used an identical recruitment approach for MSM as in a previous study in the same city, the passage of more than a decade may have led to unknown biases. HIV status was self-reported in 2023, but based on biological analysis in 2011–2012, it may be underreported in the self-report. While it is possible that increased HIV levels contributed to the increase, analysis suggested that increased levels of HIV did not play a significant role. Second, the incomplete sample, which led to the closing of the study recruitment early due to participant and staff harassment, may have provided a sample that was significantly different from the previous research on hr-HPV status. The present sample may be influenced by the addition of two additional hr-HPV genotypes (HPV-73, 82) in the 2023 analysis, although we also calculated infection by removing these two additional genotypes and found that it did not affect the number infected. Third, returning for a second vaccination dose may have been reduced by fear of harassment, despite changing the site for this dose, plus the lack of a financial incentive, and so the return rate should be regarded as being at the lower end of the range for returning for vaccination.

## 5. Conclusions

There is a high rate of anal hr-HPV in GBM in this large East African port city, appearing to increase over two decades (despite the samples being small and maybe not directly comparable). Given the high median number of HPV genotypes, high-valence vaccines and early vaccination are recommended where possible.

Despite official support for this study, social–cultural and political hostility led to study closure for safety reasons for both participants and staff. The criminalization of homosexuality poses a major challenge for vaccination and treatment for GBM specifically and for all sexual and gender minorities in SSA.

Specific strategies for vaccination and treatment of sexual and gender minorities in homophobic climates [24] should be used given legal and cultural barriers.

After the initial vaccination, over one-third returned, or would have returned if there was an incentive, for a second HPV vaccination dose. The majority were uncontactable or declined to return for a second vaccination dose. Further exploration of single-dose HPV vaccination efficacy and effectiveness in sexual and gender minority adults is warranted.

## Figures and Tables

**Table 1.** Participant characteristics.

Variable	
Age	Mean age 26.58, SD = 5.07, range 19–45
Sexual identification	
Gay men	66 (80%)
Bisexual men	14 (16%)
Transgender	3 (4%)
Annual take-home income (Tanzanian shillings)	
<50,000 TZS (about USD 20)	25 (30%)
50,001–150,000 TZS	6 (7%)
150,001–300,000 TZS	8 (10%)
300,001–450,000 TZS	4 (3%)
>450,000 TZS	33 (27%)
Did not disclose income (no response)	16 (11%)
Number of lifetime sexual partners	
1–10	28 (34%)
11–20	12 (15%)
21–100	21 (25%)
101–1000	9 (11%)
>1001	6 (7%)
Education level	
<High school	14 (17%)
Middle school	50 (60%)
High school	6 (7%)
Some college	5 (6%)
Bachelor’s degree	3 (4%)
Graduate study	2 (2%)

**Table 2.** Outcome of invitation to receive a free second HPV vaccination.

Outcome	
Received second dose in a clinic administered by a clinician	24 (28.9%)
Uncontactable via the contact method specified by them or did not return messages left	40 (48.3%)
Made appointments but did not attend	5 (6.0%)
Died (one as result of an accident, two due to HIV-related deaths)	3 (3.6%)
Required incentive to attend	8 (9.6%)
Moved out of the country	1 (1.2%)
Afraid to attend due to obvious feminization from hormone therapy	1 (1.2%)

## Data Availability

De-identified data may be available from the corresponding author.
